# *Leishmania braziliensis* Infection Enhances Toll-Like Receptors 2 and 4 Expression and Triggers TNF-α and IL-10 Production in Human Cutaneous Leishmaniasis

**DOI:** 10.3389/fcimb.2019.00120

**Published:** 2019-05-02

**Authors:** Ludmila P. Polari, Pedro Paulo Carneiro, Michael Macedo, Paulo R. L. Machado, Phillip Scott, Edgar M. Carvalho, Olívia Bacellar

**Affiliations:** ^1^Serviço de Imunologia, Complexo Hospitalar Universitário Prof. Edgard Santos, Universidade Federal da Bahia, Salvador, Brazil; ^2^Instituto Nacional de Ciência e Tecnologia de Doenças Tropicais - INCT-DT (CNPq/MCT), Salvador, Brazil; ^3^Department of Pathobiology, School of Veterinary Medicine, University of Pennsylvania, Philadelphia, PA, United States; ^4^Instituto Pesquisa Gonçalo Moniz – Fiocruz-Bahia, Salvador, Brazil

**Keywords:** human cutaneous leishmaniasis, *Leishmania braziliensis*, toll like receptor 2, toll like receptor 4, inflammation, monocytes subsets

## Abstract

Cutaneous leishmaniasis (CL) caused by infection with *Leishmania braziliensis* is characterized by an exaggerated inflammatory response that controls the parasite burden, but also contributes to pathology. While myeloid cells are required to eliminate the parasite, recent studies indicate that they may also participate in the inflammatory response driving disease progression. The innate immune response to leishmania is driven in part by the Toll-like receptors (TLRs) TLR2, TLR4, and TLR9. In this study, we used flow cytometric analysis to compare TLR2 and TLR4 expression in monocyte subsets (classical, intermediate, and non-classical) from CL patients and healthy subjects (HS). We also determined if there was an association of either the pro-inflammatory cytokine TNF or the anti-inflammatory cytokine IL-10 with TLR2 or TLR4 expression levels after *L. braziliensis* infection. *In vitro* infection with *L. braziliensis* caused CL monocytes to up-regulate TLR2 and TLR4 expression. We also found that intermediate monocytes expressed the highest levels of TLR2 and TLR4 and that infected monocytes produced more TNF and IL-10 than uninfected monocytes. Finally, while classical and intermediate monocytes were mainly responsible for TNF production, classical monocytes were the main source of IL-10. Collectively, our studies revealed that up-regulated TLR2/4 expression and TNF production by intermediate/inflammatory subsets of monocytes from patients correlates with detrimental outcome of cutaneous leishmaniasis.

## Introduction

The protozoan parasite leishmania is the causal agent of tegumentary and visceral leishmaniasis. Cutaneous leishmaniasis (CL), characterized by a well-delimited ulcer, is the most common form of American Tegumentary Leishmaniasis, and *Leishmania braziliensis* is the most important species associated with CL in the New World (Alvar et al., 2012). The immune response to *L. braziliensis* is characterized by a strong Th1 response with high production of IFN-γ, TNF and other pro-inflammatory cytokines (Bacellar et al., 2002; Gomes-Silva et al., 2007; Faria et al., 2012; Gonzalez-Lombana et al., 2013). This defense mechanism is important to control parasite growth and dissemination, but the exaggerated inflammation, mainly due the reduced ability of IL-10 to appropriately down regulate the immune response to leishmania antigens (Bacellar et al., 2002; Gonzalez-Lombana et al., 2013; Oliveira et al., 2014), contributes to the pathology of CL (Antonelli et al., 2005; Santos Cda et al., 2013; Cardoso et al., 2015; Novais et al., 2017).

Myeloid cells, including monocytes, dendritic cells, and macrophages, act as the principal host cells for *Leishmania*. Myeloid cells play a central role in the development of the immune response against these parasites via antigen presentation as well as the secretion of cytokines, chemokines, and microbicidal products. Early interactions between leishmania and macrophages can determine the outcome of the infection (Bosque et al., 2000). In *L. braziliensis* infection we have shown that macrophages from CL patients produce high amounts of TNF, CXCL9, CXCL10, and CCL3 after leishmania infection but their ability to kill the parasite is impaired (Giudice et al., 2012; Muniz et al., 2016).

Monocytes are macrophage precursors. Based on the expression of CD14 and the high affinity Fc receptor for IgG (CD16), monocytes are differentiated into three subsets: classical monocytes(CD14^high^CD16^−^), intermediate or inflammatory monocytes (CD14^high^CD16^+^), and non-classical monocytes, also known as patrolling monocytes (CD14^low^CD16^++^)(Ziegler-Heitbrock et al., 2010). Intermediate monocytes are increased in CL and are the major source of TNF, a cytokine involved in the pathology of CL (Soares et al., 2006; Passos et al., 2015). These data point to the participation of myeloid-lineage cells, in addition to T cells, in the pathology of CL.

The Toll-like receptors (TLR) are a well-characterized class of pattern recognition receptors (PRRs) and the TLR signaling pathway is one the first defense mechanisms against *Leishmania* (Medzhitov and Janeway, 2000; Tuon et al., 2008). TLRs bind to myeloid differentiation factor 88 (MyD88), resulting in downstream activation of NF-κB and the subsequent transcription of inflammatory mediators such as TNF, IL-6, and IL-1 (Medzhitov and Janeway, 2000). Macrophages recognize *Leishmania* mainly through TLR2, TLR4, and TLR9 (Becker et al., 2003; de Veer et al., 2003; Kropf et al., 2004a,b; Faria et al., 2005; Flandin et al., 2006; Viana et al., 2017).

Most of the studies about TLRs in leishmaniasis are in experimental models with different species of the parasite. For instance, C57Bl/6 MyD88-null mice are more susceptible to infection with *L. major* than wild type animals (de Veer et al., 2003) while C57BL/6J TLR2^−^/^−^ mice infected with *L. braziliensis* are more resistant to infection than C57BL/6J wild type mice (Vargas-Inchaustegui et al., 2009). Also, in C57BL/6 TLR2^−/−^ mice infected with *Leishmania amazonensis*, the parasite burden is reduced when compared with C57BL/ 6 wild type mice which were more susceptible to the infection (Guerra et al., 2010). Studies performed in BALB/c mice infected with *Leishmania donovani* showed an increase in TLR2 and TLR4 mRNA, which was correlated with parasite load (Cezário et al., 2011). In contrast, TLR4-deficient mice are unable to control *L. major* infection and develop lesions that are more severe as compared to wild type animals (Kropf et al., 2004b). Additionally, in BALB/c mice infected with *Leishmania pifanoi*, the TNF production in the infected TLR4 ^−^/^−^ bone marrow-derived macrophages was significantly lower and *in vivo* the number of parasites in footpad lesions was higher than their wild type counterpart (Whitaker et al., 2008). In CL patients, the exposure to soluble *Leishmania* antigen (SLA) enhances TLR9 expression on monocytes [30]. Moreover, the frequency of TLR9^+^ monocytes is correlated with greater lesion size (Vieira et al., 2013). However, in the lesion site, TLR9 was associated with granuloma formation (Tuon et al., 2010). These studies show that depending on the mice strain and the leishmania species, TLR expression may have either a protective or a deleterious effect on leishmania infection.

We have previously shown that *ex vivo* expression of TLR2 and TLR4 is higher on monocytes from CL patients as compared to monocytes from healthy subjects (HS) (Carneiro et al., 2016). In the present study, we investigate the expression of TLR2 and TLR4 on *L. braziliensis* infected monocyte subsets from CL patients and assess if TLR expression in monocyte subsets is associated with the production of TNF and IL-10. Our results reveal that infection with *L. braziliensis* increases the expression of TLR2 and TLR4 on inflammatory monocyte subsets and this increase is accomplished mainly by TNF production. These findings suggest that TLR expression contributes to an enhancement in the inflammatory response and pathology in the *L. braziliensis* infection.

## Materials and Methods

### Patients

A total of 30 patients with CL were included in this study. These patients sought medical attention from the Health Post of Corte de Pedra, municipality of Tancredo Neves, Bahia, Brazil, a known area of *L. braziliensis* transmission. Patients were diagnosed with CL if they presented with a clinical picture characteristic of the disease in conjunction with one of the following positive test results: parasite isolation in culture, parasite identification in histopathologic analysis, or the presence of parasite DNA by polymerase chain reaction (PCR) (Weirather et al., 2011). The CL group was composed of 25 males and 5 females. The median of age was 31 ranging between 18 and 54 years of age. A control group was formed by 20 healthy subjects (HS) living in an urban area of no exposure to leishmania, with 5 males and 15 females. The median of age was 31, ranging between 23 and 45 years.

All the experiments were performed prior to therapy. All patients were treated with i.v. meglumine antimoniate (Sanofi-Aventis, Paris, France) in a dose of 20 mg/kg body weight daily for 20 days.

### Ethics Statement

This study was carried out in accordance with the recommendations of Institutional Review Board of the Federal University of Bahia, Brazil, with written informed consent from all subjects. All subjects gave written informed consent in accordance with the Declaration of Helsinki. The protocol was approved by the Institutional Review Board of the Federal University of Bahia, Brazil (approval number 693.111).

### Human Blood Samples and Preparation of Peripheral Blood Cells

Peripheral blood mononuclear cells (PBMC) were separated from heparinized venous blood by Ficoll-Hypaque gradient centrifugation. Cells were then washed in saline and resuspended in RPMI 1640 (supplemented with 5% of fetal calf serum, 100 U penicillin/mL, 100 ug streptomycin/ mL) (GIBCO BRL., Grand Island, NY, USA).

### Parasites

An isolate of leishmania obtained from a skin lesion of a CL patient from Corte de Pedra (MHOM/BR/LTCP11245) was characterized as *L. brazilensis* using PCR and multicolus enzyme electrophoresis (Cupolillo et al., 1994). Parasites were initially grown in biphasic medium (NNN). After isolation, the parasite was cryopreserved in liquid nitrogen. The parasites selected for this study had not been previously passaged in liquid culture medium. After selection, the parasites were expanded in complete Schneider's medium (Aldrch Sigma, St. Louis, MO) supplemented with 10% fetal bovine serum (FBS) (Gilco BRL) and 2% sterile urine. For *in vitro* infection of PBMC, the promastigotes in the stationary growth phase were stained with 5 mM Carboxyfluorescein succinimidyl ester (CFSE) to identify cells infected by *L. braziliensis* (Chang et al., 2007). All the reagents and Schneider medium are endotoxin free as determined by Endotoxin Testing (LAL) (BioReliance, SIGMA-ALDRICH).

### Infection of Monocytes With *L. braziliensis*

PBMC (1 × 10^6^ cells / tube) of CL patients and healthy subjects were infected with *L. braziliensis* labeled with CFSE (as described above) at a ratio of 5:1 parasites per cell and incubated for 1 h at 37°C in a 5% CO_2_ atmosphere. After this period, extracellular parasites were washed with 0.9% saline containing 10% FBS. The cells were placed in complete RPMI 1640 medium and incubated at 37°C in an atmosphere of 5% CO_2_ for 4 h and 24 h. The infection was assessed by CFSE fluorescence (FITC) by flow cytometry.

### Expression of CD14, CD16, TLR4, and TLR2 in Monocytes From Peripheral Blood by Flow Cytometry

CL patient and healthy subject peripheral blood monocyte expression of CD14, CD16, TLR2, and TLR4 was analyzed *in vitro* after 4 h incubation with CFSE-labeled *L. braziliensis* or stimulation with one of the following reagents: lipopolysaccharide (LPS) (100 ng/ml) or synthetic TLR2 ligands tripalmitoyl-S- glycerol-Cys-(Lys)4 (Pam3Cys) (100 ng/ml). The analysis was performed by flow cytometry. The following antibodies were used: anti-CD14 conjugated with PerCP-Cy5.5 (clone 61D3) and anti-CD16 conjugated to APC (clone CB16) (eBioscience, San Diego, CA, USA); anti-TLR2 conjugated to PE (clone TL2.1) and anti-TLR4 PE-conjugated (clone HTA125) (IMGENEX, San Diego, CA, USA). Analysis of TLR2 and TLR4 expression was undertaken in separate tubes. After staining, cells were washed and resuspended in 4% paraformaldehyde solution. We acquired at least 200,000 events on the flow cytometry BD FACS CANTOII. Data analysis was performed using FlowJo software (Free Star Inc.).

### Analysis of the Expression of TNF and IL-10 in Monocytes by Flow Cytometry

PBMCs were either infected with parasites of *L braziliensis* or left uninfected. Infected and uninfected PBMCs were then incubated separately for 8 h and 24 h at 37°C, 5% CO_2._ Cells were then stained with anti-CD14 monoclonal antibodies (PerCP Cy-5.5), anti-CD16 (APC), anti-TLR2 (PE) and anti-TLR4 (PE) for 15 min 4°C in the dark (BD-Bioscience). The cells were washed with PBS (1,500 rpm, 5 min, 4°C), fixed with 4% paraformaldehyde, and permeabilized with Perm Wash solution for 15 min at 4°C in the dark (BD-Bioscience). Intracellular staining was performed with anti-TNF and anti-IL-10 (FITC) antibodies for 30 min. After this period, the cells were washed and suspended in 400 μl PBS for flow cytometry analysis on BD FACS CANTOII. A total of 200,000 events were acquired. Data analysis was performed using FlowJo program (Free Star Inc.).

### Statistical Analysis

Data analyses were performed using GraphPad Prism 5.0 (GraphPad Software, Inc., San Diego, CA, USA). The comparison between groups was performed using the non-parametric Mann-Whitney U test. Analysis of variance (Kruskal-Wallis) was calculated to assess the differences between three or more groups, with Dunn's post-test. Analysis of variance (ANOVA) with Bonferroni post-test's was performed when the data presented normal Gaussian distribution. An error below 5% (*p* < 0.05%) was used for statistical significance.

## Results

### *Ex vivo* Expression of TLR2 and TLR4 on Different Monocytes Subsets

**Figure 1:**
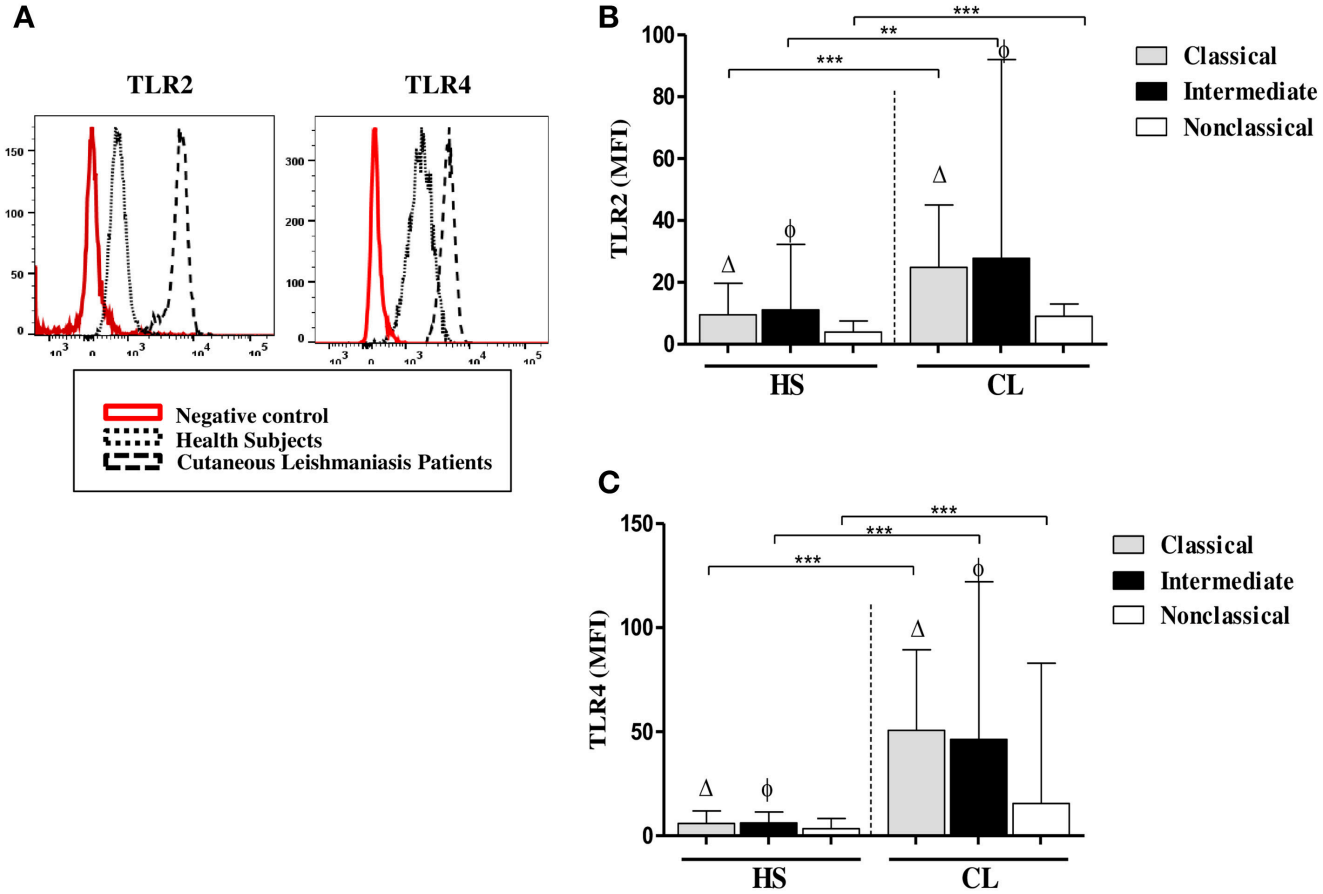
Classical (CD14^high^CD16^−^) and intermediate monocytes (CD14^high^CD16^+^) express more TLR2 and TLR4 than non-classical (CD14^low^CD16^+^) monocytes. **(A)** Representative strategy analysis for the TLR2 and TLR4 expression. **(B)** *Ex vivo* expression of TLR2 on monocytes subsets from CL patients and HS group (*n* = 10). **(C)** *Ex vivo* expression of TLR4 in monocytes subsets from CL patients and HS group (*n* = 10). Data are represented by the median of the mean intensity of fluorescence (MIF).Mann-Whitney and Kruskal-Wallis with Dunn's post-test was used for statistical analyses. (Δ) classical vs. non-classical monocytes, (φ) intermediate vs. non-classical monocytes, (^**^P < 0.01, ^***^P < 0.001).

We had shown that e*x vivo* expression of TLR2 and TLR4 on monocytes from CL patients was higher than on monocytes from HS (Carneiro et al., 2016). Monocytes are a heterogeneous population of cells and there are three monocytes subsets based on the expression of CD14 and CD16. The expression and CD14 CD16 was not modified after infection with *L.braziliensis* (data not shown) but it known that the frequency of intermediate (inflammatory) monocytes is higher in CL patients than in HS (Soares et al., 2006; Passos et al., 2015). In the present study, to determine if the expression of TLRs differs among monocyte subsets, we analyzed the *ex vivo* expression of TLR2 and TLR4 on classical, intermediate, and non-classical monocytes (Figure 1). In CL patients, TLR2 expression, represented by the mean fluorescence intensity (MFI), was more intense in classical and intermediate monocytes than in non-classical monocytes, although only the latter achieved statistical significance, *p* < 0.001 (Figure 1B). The MFI for TLR4 (Figure 1C) in CL patients was similar in classical and intermediate monocytes and both subpopulations expressed more TLR4 than non-classical monocytes (*p* < 0.01). In HS, the MFI for TLR2 and TLR4 was higher in classical and intermediate monocytes than in non-classical monocytes (Figures 1B,C). In HS group, the expression of these receptors in all monocytes subsets was lower than in CL monocytes (*p* < 0.01 and *p* < 0.001). A comparative analysis of the MFI for TLR2 and TLR4 in CL patients vs. HS in intermediate monocytes showed that cells from CL patients expressed more TLR2 and TLR4 than cells from the HS group (*p* < 0.001).

### Infection With *L. braziliensis* Increase TLR2 and TLR4 Expression on Monocytes From CL Patients

**Figure 2:**
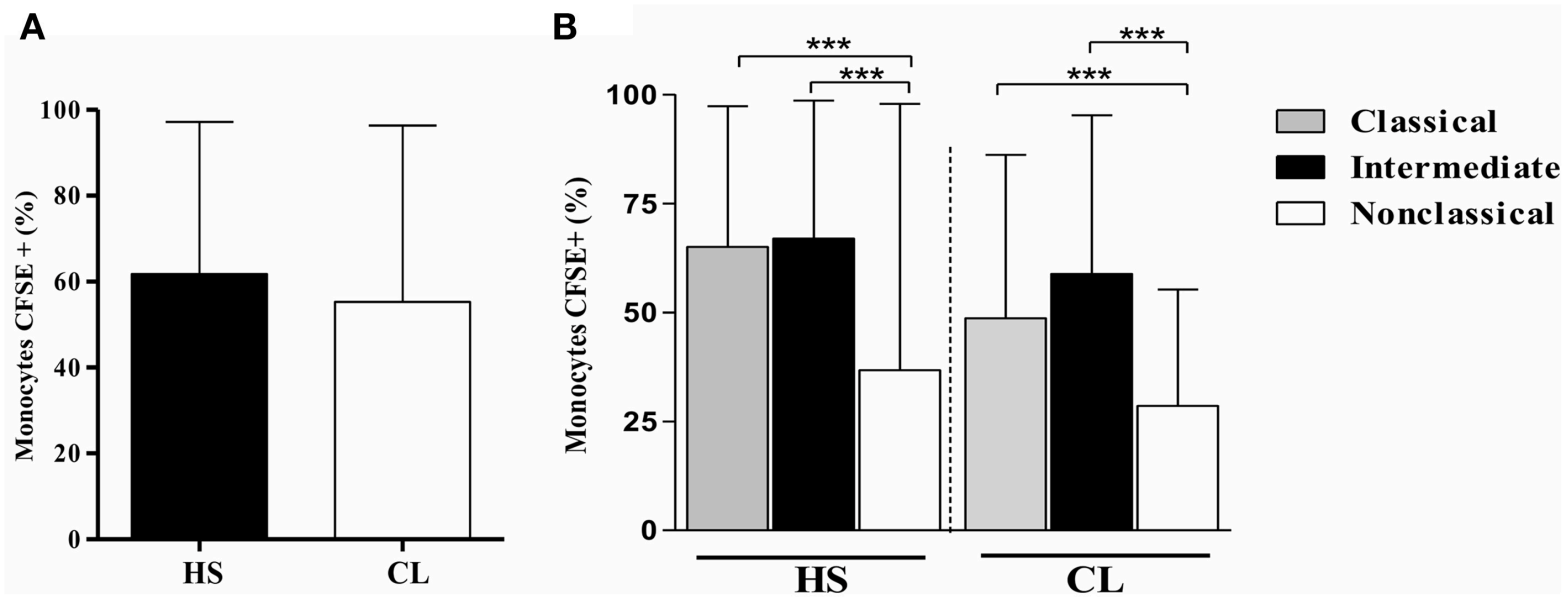
The frequency of *L. braziliensis*-infected monocytes is similar in CL and HS cells. Classical and intermediate monocytes are more infected than non-classical monocytes in CL and HS groups. **(A)** Frequency of PBMC-derived monocytes (CD14^+^CD16^+^) from CL patients (*n* = 8) and HS (*n* = 8) infected with CFSE-labeled parasites after 4 h of culture. **(B)** Frequency of infected classical (CD14^high^CD16^−^), intermediate (CD14^high^CD16^+^), and non-classical monocytes (CD14^low^CD16^+^) from CL patients and HS after 4 h of culture. Data are represented by the median of the frequency of cells infected. Kruskal-Wallis with Dunn's post-test was used for statistical analyses (^***^P < 0.001).

To assess if the infection with *L. braziliensis* modifies either TLR2 or TLR4 expression in CL monocytes, the expression of these receptors was evaluated on *L. braziliensis-*infected monocytes. First, the infection rate on monocytes from CL and HS was compared. The frequency of *L. braziliensis*-infected monocytes was similar in CL cells and in HS cells, 55and 62%, respectively, *p* > 0.05 (Figure 2A). Also, we found that classical and intermediate monocytes were more infected than non-classical monocytes and there was no difference in CL and HS (Figure 2B)

**Figure 3:**
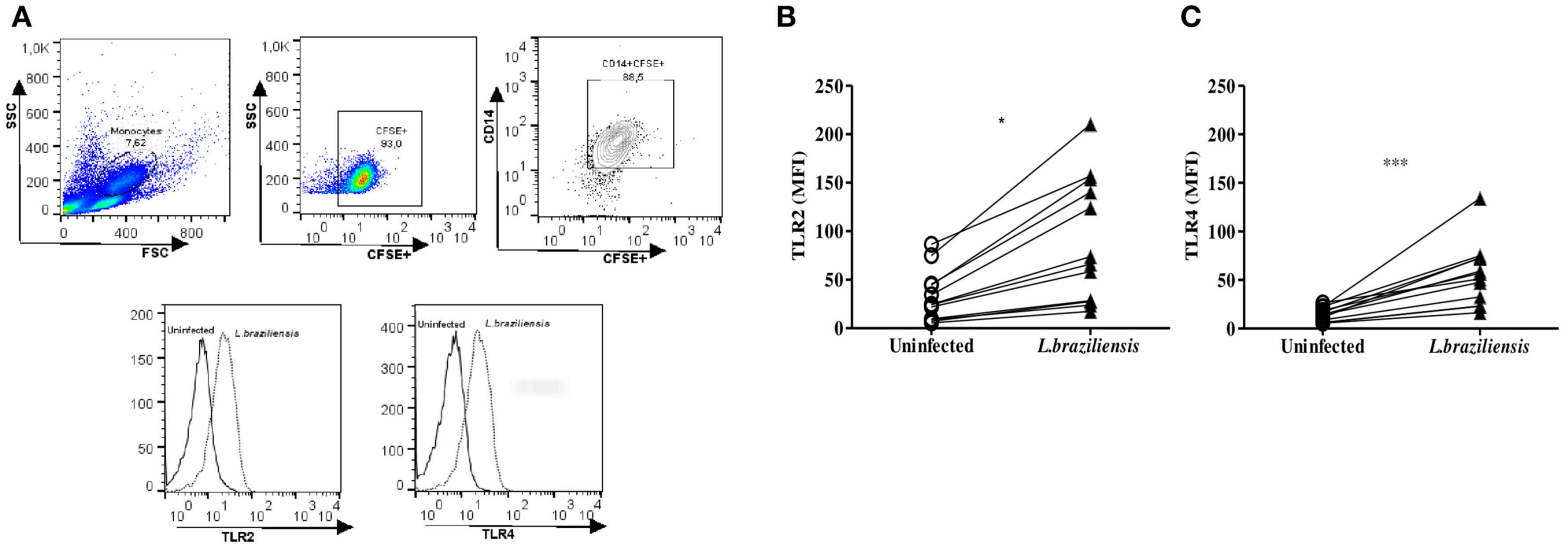
*L. braziliensis* up regulate the expression of TLR2 and TLR4 on monocytes from CL patients. PBMC-derived monocytes from CL patients (*n* = 08) and HS (*n* = 08) were infected for 4 h with *L. braziliensis* (ratio 5:1) stained with CFSE. **(A)** Representative strategy analysis for the TLR2 and TLR4 expression after infection by *L. braziliensis* **(B)** TLR2 and **(C)** TLR4 expression in monocytes from CL after infection with *L. braziliensis*. Data are represented by the median of the mean intensity of fluorescence (MIF). Wilcoxon test were used for statistical analyses (^*^P < 0.05, ^***^P < 0.01).

Next, the expression of these receptors was evaluated on non-infected cells and *L. braziliensis*-infected cells from CL patients. The median MFI of TLR2 and TLR4 on infected monocytes was significantly higher (*p* < 0.001) than that observed in uninfected monocytes (Figures 3A,B). We also evaluated the expression of TLR2 and TLR4 on monocytes from HS after infection with *L.braziliensis*. The infection with *L.braziliensis* increased the expression of TLR2 and TLR4 on monocytes from HS. However, the expression of TLR2 and TLR4 was lower than that observed in CL patients, 25 (22-29); (Whitaker et al., 2008; Guerra et al., 2010; Cezário et al., 2011) vs. 70 (27–150, < 0.05) and 43 (24–56) vs. 54 (25–72, *p* < 0.05), respectively. There was no difference in the expression of these receptors between the different periods of infection in both groups, CL and HS (Supplementary Figure 1).

### TLR2 and TLR4 Expression on Different Monocytes Subsets After Infection With *L. braziliensis*

Because the *ex vivo* expression of these receptors was higher on inflammatory monocytes from CL patients, further experiments evaluating the expression of TLR2 and TLR4 on monocyte subsets after infection with *L. braziliensis* were performed.

**Figure 4:**
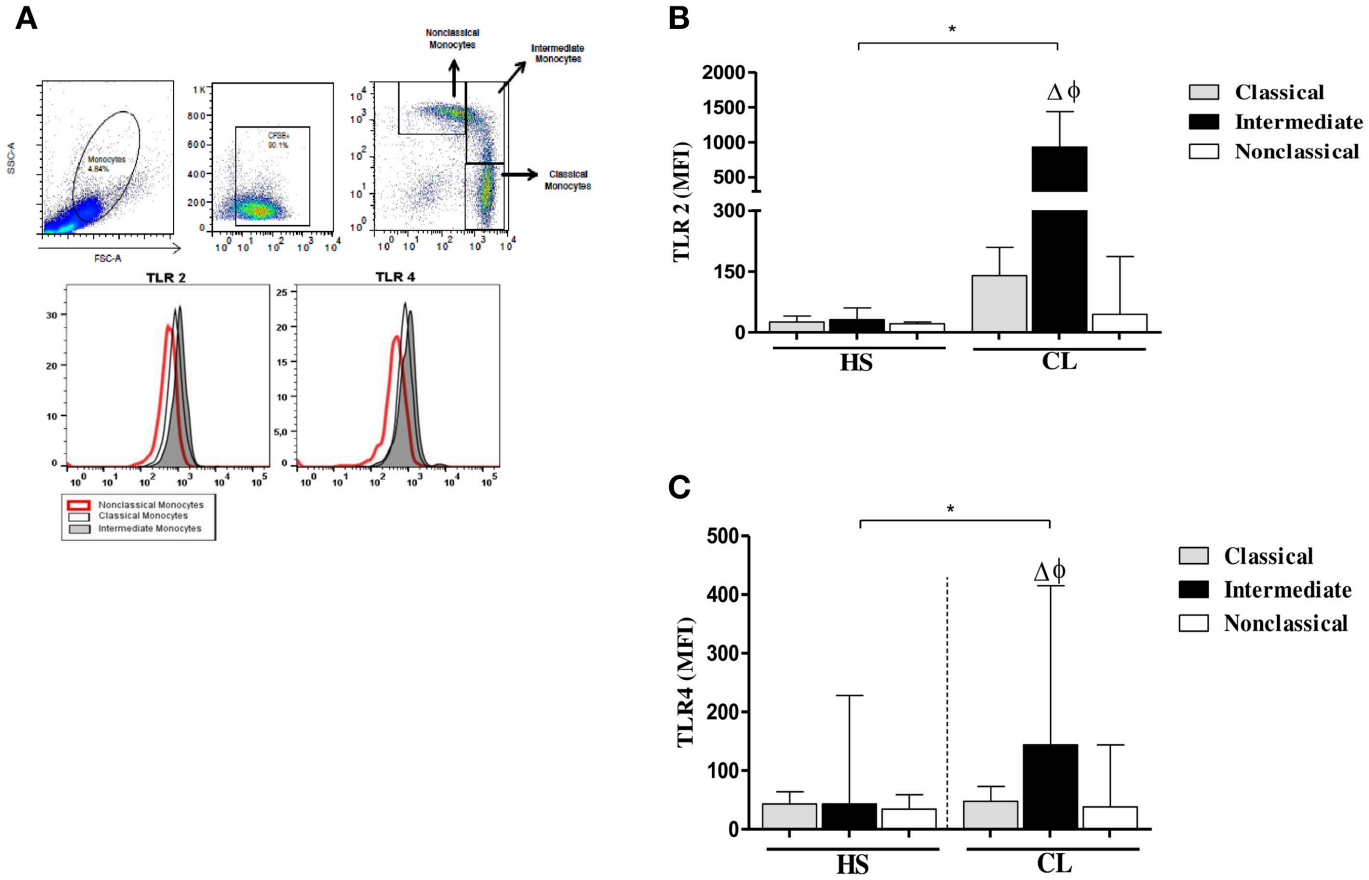
TLR2 and TLR4 expression in monocytes subsets from CL patients after infection by *L. braziliensis*. PBMC-derived monocytes from CL patients (*n* = 08) and HS (*n* = 08) were infected for 4 h with *L.braziliensis* (ratio 5:1) stained with CFSE for 4 h. **(A)** Representative strategy analysis for the selection of monocytes subsets and TLR2 and TLR4 expression. **(B)** TLR2 expression in classical (CD14^high^CD16^−^), intermediate (CD14^high^CD16^+^), and non-classical monocytes (CD14^low^CD16^+^) from CL patients and HS after infection by *L. braziliensis*.**(C)** TLR4 expression in monocytes subsets from CL patients and HS after infection by *L. braziliensis*. Data are represented by the median of the mean intensity of fluorescence (MIF). (Δ) Intermediate vs. classical monocytes, *P* < 0.01 (φ) Intermediate vs. non-classical monocytes, *P* < 0.01. Mann-Whitney and Kruskal-Wallis with Dunn's post-test was used for statistical test was used for statistical analyses (^*^P < 0.05).

The intensity of expression of TLR2 and TLR4 on different monocyte subsets from CL patients and HS after infection with *L. braziliensis* is shown in Figure 4.

In fact, the infection with *L. braziliensis* increased the expression of TLR2 and TLR4 on intermediate monocytes. Again, in CL patients, TLR2 expression was more intense in classical and intermediate monocytes than in non-classical monocytes (Figure 4B), Also, the expression of TLR4 was higher on intermediate monocytes than in classical and non-classical monocytes. However, the expression of TLR2 and TLR4 on *L. braziliensis* infected intermediate monocytes from CL patients was higher than that observed on monocyte subsets from HS individuals (Figures 4A,B). In uninfected monocytes the expression of these receptors was lower than in infected cells in both groups. Additionally, we evaluate the expression of TLR2 and TLR4 after 24 h of infection and the expression of these receptors was similar to that obtained after 4 h of infection. The expression of these receptors was also higher in intermediate monocytes (Supplementary Figure 2).

So far, these results indicate that in human CL, in addition to TLR2 and TLR4 are preferentially expressed in intermediate monocytes, the infection with *L. braziliensis* increases the expression of these receptors on this monocyte subset. Thus, increased expression of TLR2 and TLR4 in the CL patient's intermediate monocytes may result in an enhancement of the inflammatory response.

### Evaluation of the Intracellular Expression of TNF and IL-10 in Monocytes From CL Patients Expressing TLR2 and TLR4 After Infection With *L. braziliensis*

**Figure 5:**
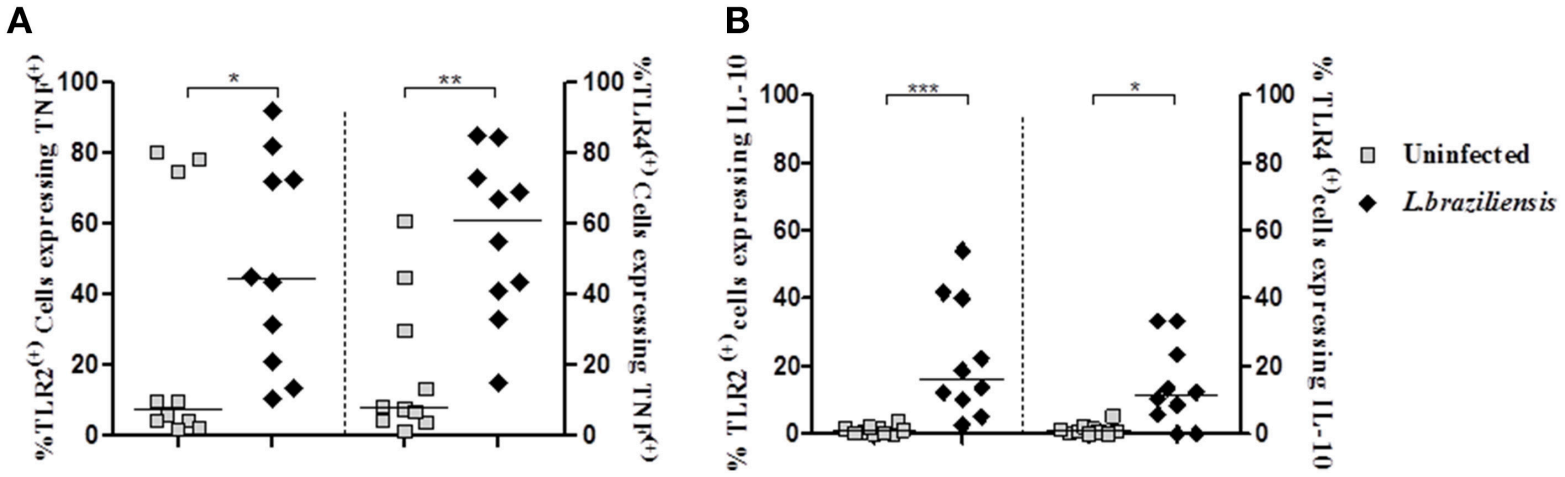
Infection with *L. braziliensis* increases the frequency of TLR2 ^(+)^ and TLR4 ^(+)^ monocytes expressing TNF and IL-10. PBMC-derived monocytes from CL patients (*n* = 9) were infected with *L. braziliensis* (ratio 5:1) for 8 h. The data represent the frequency of TLR2 ^(+)^ and TLR4 ^(+)^ on monocytes (CD14+) from CL patients (*n* = 9) expressing TNF **(A)** and IL-10 **(B)**. The percentage of CD14+ cells expressing cytokines in non-infected and infected cells was determined by gating on the corresponding population. The results are expressed as median and Mann-Whitney test was used for statistical analyses (^*^P < 0.05, ^**^P < 0.01, ^***^P < 0.001).

TLRs initiate innate immune responses in a variety of ways, leading to the production of inflammatory cytokines, such as TNF, by dendritic cells, macrophages, and monocytes (Kuniyoshi et al., 2014). IL-10 is the most important regulatory cytokine in leishmaniasis (Carvalho et al., 1994b; Bomfim et al., 1996; Bacellar et al., 2002). The high levels of TNF and the decreased ability of IL-10 to down regulate cytokine production leads to an exacerbation of the inflammatory reaction and development of cutaneous and mucosal leishmaniasis following infection with *L. braziliensis* (Da-Cruz et al., 1996; Bacellar et al., 2002; Antonelli et al., 2005). Thus, we asked if the increased expression of TLR2 and TLR4 on infected-monocytes was associated with an increase in production of TNF and IL-10 by these cells. While the frequency of infected monocytes co-expressing TLR2 and TNF was 44% (19–74%) and TLR4 and TNF was 61% (39–75%), in non-infected monocytes co-expression of these receptors and TNF was 7% (3–75%) and 7% (4–33%), respectively (Figure 5A). Similar data was observed regarding IL-10. The frequency of cells expressing IL-10 in TLR2 ^(+)^ and TLR4 ^(+)^ infected monocytes was higher than that observed in non-infected monocytes expressing these receptors (Figure 5B). These data reveal that expression of TLR2 and TLR4 up regulate TNF and IL-10 expression in infected monocytes from CL patients.

### TNF and IL-10 Are Expressing Mainly in TLR2 ^(+)^ and TLR4 ^(+)^ Infected Monocytes

Due to the observation that TNF and IL-10 expression was enhanced in TLR2 ^(+)^ and TLR4 ^(+)^ infected cells, we evaluated the expression of TNF and IL-10 in TLR ^(+)^ and in TLR ^(−)^ infected monocytes in order to investigate if these cytokines are preferentially expressed on TLR2 ^(+)^ and TLR4 ^(+)^ infected monocytes.

**Figure 6:**
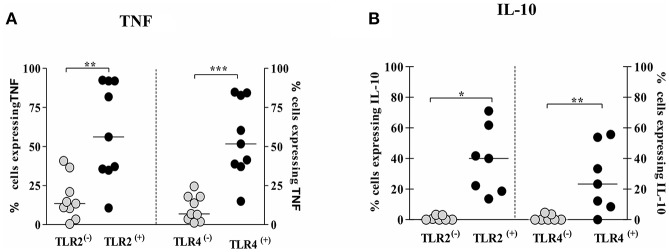
Monocytes from CL patients expressing TLR2 and TLR4 produce more TNF and IL-10 after *L.braziliensis infection*. PBMC-derived monocytes from CL patients (*n* = 9) were infected with *L.braziliensis* (ratio 5:1) for 8 h. The data represent the frequency of cells expressing TNF **(A)** and IL-10 **(B)** on monocytes TLR^(+)^ or TLR^(−)^. The frequency of CD14+ cells expressing cytokines on cells TLR^(+)^ or TLR^(−)^ was determined by gating on the corresponding population. The results are expressed as median and Mann-Whitney test was used for statistical analyses (^*^P < 0.05, ^**^P < 0.01, ^***^P < 0.001).

The frequency of cells expressing intracellular TNF was higher in TLR2 ^(+)^ and TLR4 ^(+)^ infected monocytes, 44%(19–74%) and 61%(39–75%), respectively, than TLR2 ^(−)^ and TLR4 ^(−)^ infected monocytes, 13%(7–28%) and 7%(3–17%), respectively (Figure 6A). Similarly, the frequency of cells expressing intracellular IL-10 was higher on TLR2 ^(+)^ and TLR4 ^(+)^ infected monocytes than on TLR2 ^(−)^ and TLR4 ^(−)^ infected monocytes (Figure 6B). In a small number of patients we also evaluate the expression of these cytokines after 24 h of infection and as expected there was a decrease in the intracellular expression of TNF and IL-10 as these cytokines are predominantly detected early after infection (Supplementary Figure 3).

Together, these data reinforce the idea that the up regulation of these receptors, after infection with leishmania, activates the monocytes to produce TNF and IL-10.

### Intracellular Expression of TNF and IL-10 on Monocytes Subsets From CL Patients After Infection With *L. braziliensis*

The classical CD14^++^CD16^−^ monocytes specialize in phagocytosis, production of reactive oxygen species, and secretion of IL-10, CCL2, IL-6, and TNF in response to ligands for extracellular TLRs (such as the bacterial product LPS) (Saha and Geissmann, 2011; Wong et al., 2012). The intermediate subset displays the characteristics of activated cells. Such cells have elevated intracytoplasmic levels of pro-inflammatory cytokines such as TNF (Hristov and Weber, 2011; Wong et al., 2012).

**Figure 7:**
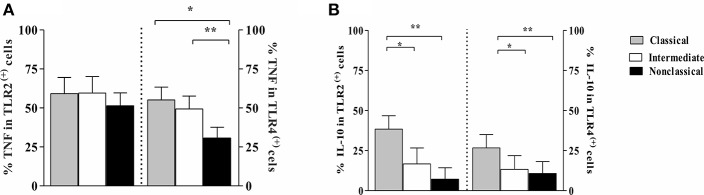
Frequency of monocytes subsets from CL patients expressing TNF and IL-10 in cells TLR2 ^(+)^ and TLR4 ^(+)^ after *L. braziliensis infection*. PBMC-derived monocytes from CL patients (*n* = 9) were infected with *L. braziliensis* (ratio 5:1) and stained with CFSE for 8 h. The data represent the frequency of cells expressing TNF **(A)** and IL-10 **(B)** on classical (CD14^high^CD16^−^), intermediate (CD14^high^CD16^+^), and non-classical monocytes (CD14^low^CD16^+^) after infection with *L. braziliensis*. The percentage of monocytes expressing cytokines on cells TLR ^(+)^ was determined by gating on the corresponding population. The results are expressed as median. Kruskal-Wallis with Dunn's post-test were used for statistical analyses (^*^P < 0.05, ^**^P < 0.01).

Because we observed that intermediate monocytes express more TLR2 and TLR4 than classical and non-classical monocytes after infection with leishmania, we analyzed the frequency of cells expressing TNF and IL-10 on monocyte subsets expressing TLR2 and TLR4. Figure 7 shows the percentage of cells within each monocyte subset that express these cytokines. There was no difference in TNF expression on monocyte subsets expressing TLR2 (Figure 7A). However, the percentage of TLR4 ^(+)^ classical and intermediate monocytes expressing TNF was higher than TLR4 ^(+)^ non-classical monocytes. Moreover, TLR2 ^(+)^ and TLR4 ^(+)^ classical monocytes were the main source of IL-10 (Figure 7B).

We recognized that experimental design adopted in this study may simulate the scenario of reinfection as the patients remained in the endemic area and could be continually exposure to sandflies bites. In such case, the *ex vivo* expression of TLR2 and TLR4 and cytokines spontaneously produced by the intermediate monocytes could also reflect stimulation of NLRP3 inflammasome by pro-inflammatory molecules derived from sandflies derived-microbiota (Dey et al., 2018).

## Discussion

The TLRs are a well-characterized class of pattern recognition receptors that are expressed on phagocytes and interact with PAMPs expressed on the surface of infectious agents (Ozinsky et al., 2000). TLR activation by parasite molecules trigger nuclear factor the nuclear localization of transcription factor NF-κB and mitogen activated protein kinase (MAPk) signaling pathways, to induce expression of pro-inflammatory cytokines genes that are essential for controlling parasite replication (Tuon et al., 2008). However, in several infectious diseases, such as tuberculosis, malaria, and toxoplasmosis, TLR2 and TLR4 have been considered important in the development of the inflammatory response and pathology (Mukherjee et al., 2016). We have previously described that *ex vivo* expression of TLR2 and TLR4 was higher on monocytes from CL patients than on HS cells (Carneiro et al., 2016). In this study we show that the expression of these receptors was higher on classical and intermediate monocytes from CL patients. The expression of TLR2 and TLR4 was higher on intermediate monocytes from CL patients than on cells from the HS group. We also demonstrate that following *in vitro* infection with *L. braziliensis*, TLR2 and TLR4 expression is up-regulated in cells from CL patient monocytes as compared to HS monocytes.

On cells from CL patients the expression of TLR2 and TLR4 was highest on classical and intermediate monocyte subsets. As the infection rate was similar between monocyte subsets from CL and HS (Figure 2A), the up regulation of these receptors on cells from CL after infection with *L. braziliensis* suggests that peripheral blood monocytes are already activated and the interaction with the parasite induces the increased expression of these receptors. Also, the increased expression of TLRs may be due to recognition of *Leishmania* lipophosphoglycans (LPGs) by the innate immune system (Tuon et al., 2008). *L. braziliensis* LPG is a strong agonist of TLR2, inducing TNF, IL-1β, and IL-6 production (Ibraim et al., 2013). Our results give support to the finding of increased expression of TLR2 and TLR4 in skin lesions from CL patients caused by *L.braziliensis* (Campos et al., 2018).

Previous studies have evaluated the importance of the TLR in mice infected with leishmania, but the functional role of TLRs in human leishmaniasis still needs to be elucidated. TLR signaling has been linked to pro-inflammatory responses (Medzhitov and Janeway, 2000). For instance, macrophages from MyD88^−^/^−^TRIF^−^/^−^ *L. panamensis* infected C57BL/6 mice, which are unable to activate TLR-dependent pathways, have a decreased ability to secrete TNF and an increased parasite burden early in the infection (Gallego et al., 2011). In contrast, in TLR2-deficient C57BL/6 mice infected with *L. amazonensis*, a decrease in parasitic load and in the recruitment of inflammatory cells at the infection site was observed in the early stages of infection, suggesting that absence of this receptor decreases inflammation and favors the control of parasitic burden (Guerra et al., 2010). The results found in the studies involving TLR4 are also controversial. TLR4-deficient C57BL/10ScN mice are more susceptible to *L. major* infection, presenting with more severe lesions and higher parasitic load than TLR4-competent mice, an observation that was associated with an increase in IL-10 synthesis and IL-4 receptor expression (Kropf et al., 2004a). However, macrophages from TLR4^−^/^−^ C57BL/6 infected with *L. panamensis* are able to clear amastigotes (Gallego et al., 2011).

IFN-γ is the main cytokine that activates macrophages to kill parasites. However, clearance of the leishmania parasite is also mediated by TNF. Monocytes are the main source of TNF and the importance of this cytokine in the pathology of CL and ML caused by *L. braziliensis*, has been well documented (Lessa et al., 2001; Antonelli et al., 2005; Oliveira et al., 2014; Passos et al., 2015).

To further assess the potential roles of TLR signaling and cytokine production by distinct monocyte subsets in *L. braziliensis* infection, we evaluated their expression in CL monocytes before and after infection with *L. braziliensis*. First, we showed that after infection with *L. braziliensis* there is an increase in TNF expression and it occurs predominantly in TLR2 ^(+)^ and TLR4 ^(+)^ cells. Giving support to the role of TLR4 in cytokine secretion, Galdino et al. demonstrated that infection with *L. braziliensis* increases the production of TNF and IL-10 by human cells in a TLR4 dependent manner (Galdino et al., 2016). However, a small number of both TLR ^(−)^ and uninfected monocytes also expressed TNF. This observation is likely due to the ability of *L. braziliensis* infected cells to induce TNF production in uninfected bystander cells (Carvalho et al., 2008).

Previously, we have shown that while classical monocytes have the ability to kill leishmania, intermediate monocytes were the main source of TNF (Novais et al., 2014; Passos et al., 2015). Here we demonstrated the importance of TLR2 and TLR4 in cytokine secretion and that in addition to intermediate monocytes, classical monocytes expressing TLR4 also produce TNF. Moreover, while all monocyte subsets express TNF, classical and intermediate monocytes expressing TLR4 were the main source of this cytokine. We also show that TLR2 ^(+)^ and TLR4 ^(+)^ cells express TNF and IL-10. As more than 60% of TLR2 ^(+)^ or TLR4 ^(+)^ cells expressed TNF and a large percentage of TLR ^(+)^ monocytes also expressed IL-10, it is likely that some cells express both inflammatory and anti-inflammatory cytokines.

IL-10 is the major regulatory cytokine in human leishmaniasis (Carvalho et al., 1994a; Bacellar et al., 2002; Gautam et al., 2011). Although IL-10 is associated with parasite persistence and dissemination (Bomfim et al., 1996; Anderson et al., 2008), it is also important for controlling the exaggerated inflammatory response associated with pathology observed in parasitic diseases such as malaria, Chagas disease, and leishmaniasis (Li et al., 2003; Costa et al., 2009, 2015; Gautam et al., 2011). Classical monocytes are the main source of IL-10 after stimulation with LPS (Wong et al., 2011). Therefore, the increased expression of IL-10 in classical monocytes may have two explanations. As classical monocytes are cells responsible for leishmania killing (Novais et al., 2013), the increase in IL-10 may be one way leishmania is able to escape host defense mechanisms. Alternatively, IL-10 production may represent an attempt of the classical monocytes to attenuate pathology mediated by the exaggerated pro-inflammatory response of the intermediate monocyte (Cyktor and Turner, 2011).

As the experiments in this study were performed with promastigotes in the stationary phase and it known that this population contain about 20% of the parasites that are not metacyclics promastigotes (Viana et al., 2017), there is a minor chance that the results obtained with such global parasite populations might be shaped by pro-inflammatory molecules produced by the stationary promastigotes, rather than from the 80% metacyclics parasites.

While TLRs participate in host defense mechanisms by promoting secretion of pro-inflammatory molecules and development of a Th1 type immune response, their role in the pathology of human CL has not been clearly documented. Comparing expression of TLR2 and TLR4 in macrophages from patients infected with *L. major*, Tolouei et al. showed that on macrophages from patients who had healing lesions with no history of treatment, TLR2 and TLR4 expression was higher than macrophages from CL patients with non-healing lesions and with an illness duration of more than 1 year (Tolouei et al., 2013). While this finding suggests that a decrease in TLR expression may impair the control of the infection, the patients evaluated in this study with no healing lesions had history of at least two full courses of treatment with Glucantime which could explain the decreased expression of these receptors on cells from these patients. Here, we showed the importance of TLR2 and TLR4 expression in the production of TNF, a cytokine associated with pathology in human CL caused by *L. braziliensis*.

We add to the body of knowledge about TLRs in *L. braziliensis* infection and about different monocyte subset functions. *L. braziliensis* infection enhanced TLR2 and TLR4 receptors as well as the frequency of classical and intermediate monocytes expressing these receptors. Moreover, while classical and intermediate monocytes expressing TLR2 and TLR4 are the main cells secreting TNF, the classical monocytes are the major cell source of IL-10. This study also has implications in immunotherapy for infectious diseases. As TLRs trigger inflammatory responses, agonists of TLRs have been used as adjuvants in vaccines against leishmania infection in experimental animals (Calvopina et al., 2006; Raman et al., 2010). However, our data show that *L. braziliensis* enhances TLR2 and TLR4 which leads to a pro-inflammatory environment that does not prevent the appearance of the disease. Thus, it is possible that over expression of TLRs may be more related to pathology than protection in human CL. As TLR antagonist molecules have been used in the treatment of inflammatory diseases (Gao et al., 2017), studies evaluating the role of TLR2 and TLR4 antagonists in the modulation of the inflammatory response in patients with CL should be performed.

## Ethics Statement

This study was carried out in accordance with the recommendations of Institutional Review Board of the Federal University of Bahia, Brazil, with written informed consent from all subjects. All subjects gave written informed consent in accordance with the Declaration of Helsinki. The protocol was approved by the Institutional Review Board of the Federal University of Bahia, Brazil (approval number 693.111)

## Author Contributions

LP, PC, and OB participated equally in the study design and in the writing of the manuscript. LP and PC participated equally in all experiments. MM participated in the human macrophages infection and processing of samples on the flow cytometer. PM is a dermatologist and participated in the diagnostic of the patients in the endemic area and in the discussion of the results. EC and OB are the principal investigators of this work and followed the work from the beginning to the end and also participated in the writing of the manuscript. PS participated in the discussion of the results and in the writing of the manuscript.

### Conflict of Interest Statement

The authors declare that the research was conducted in the absence of any commercial or financial relationships that could be construed as a potential conflict of interest.
