# Supplementary figures and images for: Leishmania braziliensis Infection Enhances Toll-Like Receptors 2 and 4 Expression and Triggers TNF-α and IL-10 Production in Human Cutaneous Leishmaniasis
Source: Front Cell Infect Microbiol. 2019 May 2;9:120. doi: 10.3389/fcimb.2019.00120 (PMC6507514; doi:10.3389/fcimb.2019.00120)

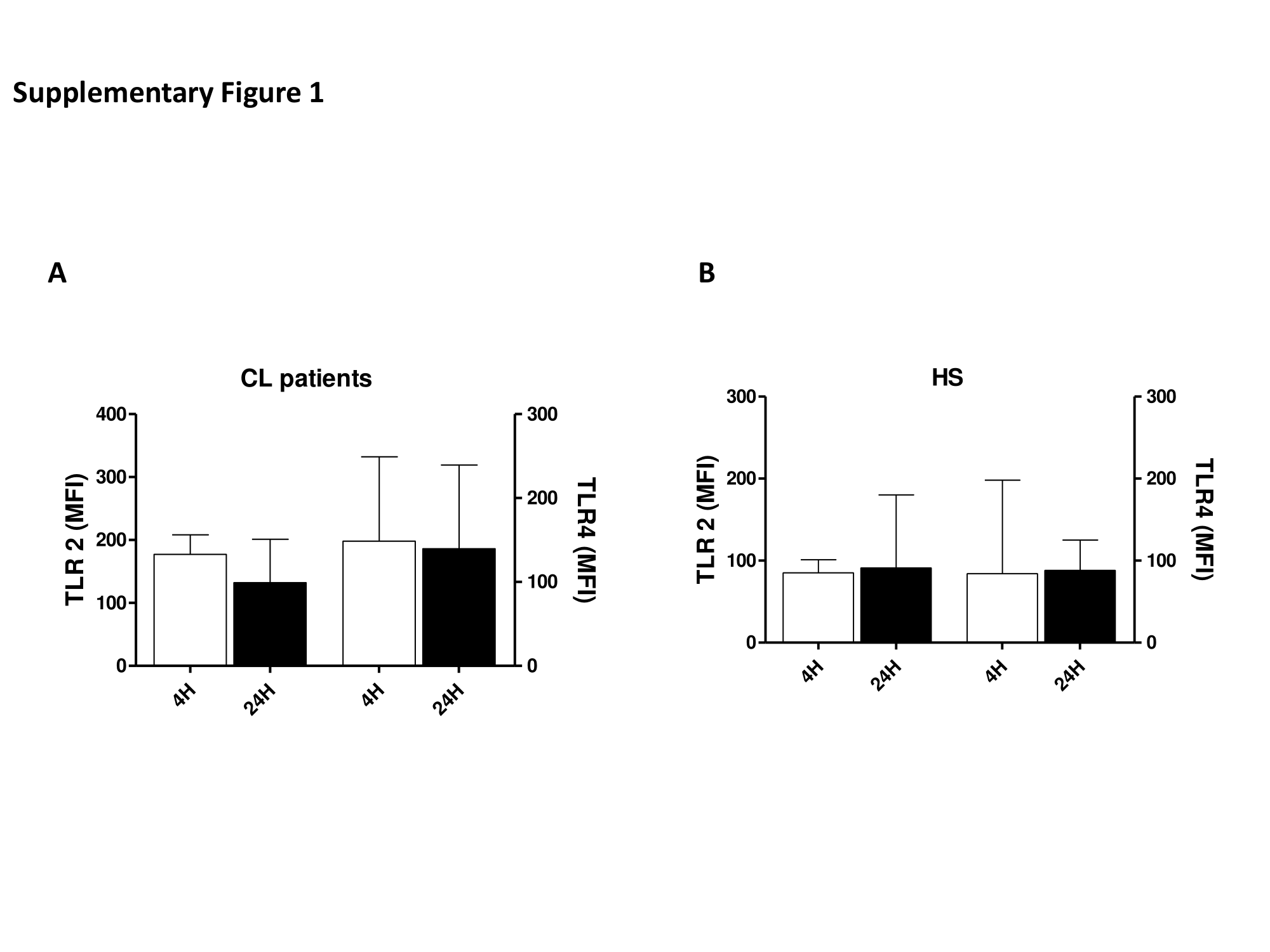

Supplement: Supplementary file 1 [file Image_1.tiff]

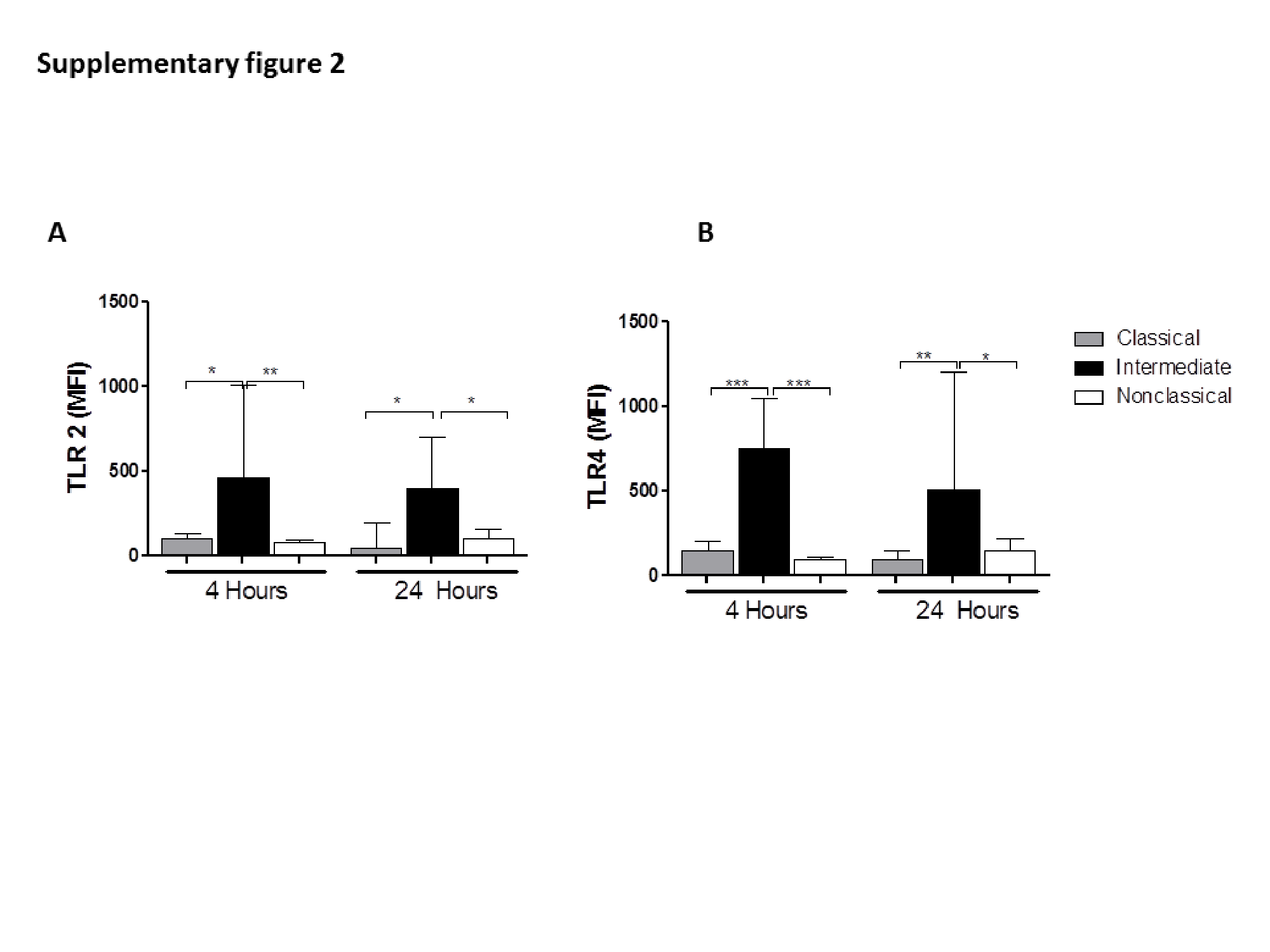

Supplement: Supplementary file 2 [file Image_2.tiff]

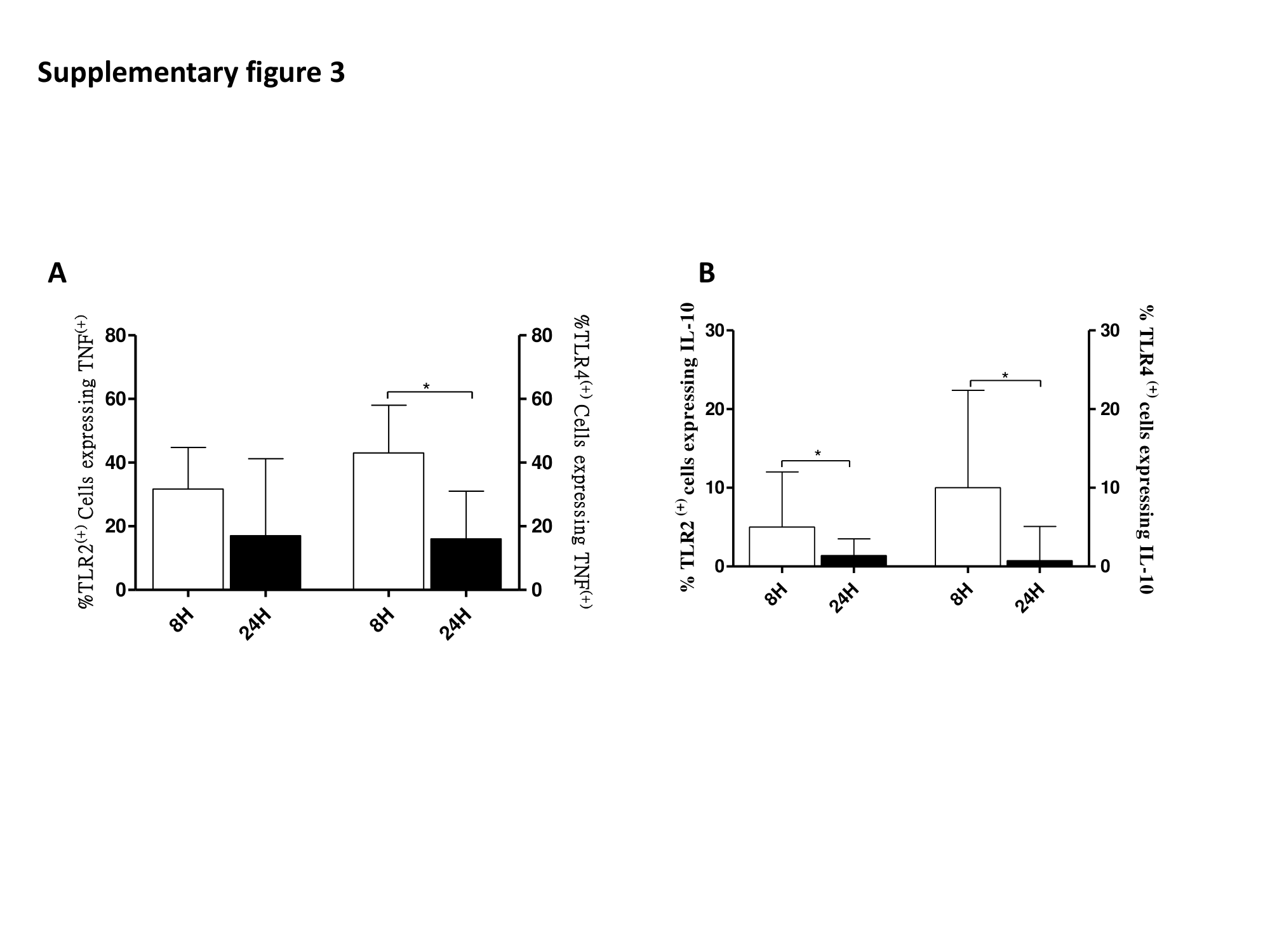

Supplement: Supplementary file 3 [file Image_3.tiff]
