# Supplementary material for: Leishmania braziliensis Infection Enhances Toll-Like Receptors 2 and 4 Expression and Triggers TNF-α and IL-10 Production in Human Cutaneous Leishmaniasis
Source: Front Cell Infect Microbiol. 2019 May 2;9:120. doi: 10.3389/fcimb.2019.00120 (PMC6507514; doi:10.3389/fcimb.2019.00120)
Supplement: Supplementary file 4 [file Image_4.pdf]

Supplementary figure 1. Expression of TLR2 and TLR4 in Monocytes from Cutaneous Leishmaniasis Patients (CL) and Healthy Subjects (HS) after infection with *L.braziliensis* for 4 and 24 hours. PBMC-derived monocytes from CL patients (n=05) and HS (n=05) were infected for 4 and 24 hours with *L. braziliensis* (ratio 5:1) stained with CFSE. **(A)** Expression of TLR2 and TLR4 in monocytes from CL patients **(B)** Expression of TLR2 and TLR4 in monocytes from HS. Data are represented by the median of the mean intensity of fluorescence (MIF). Mann Whitney test were used for statistical analyses.

Supplementary figure 2. Expression of TLR2 and TLR4 in Monocytes Subsets from Cutaneous Leishmaniasis patients (CL) after infection with *L.braziliensis* for 4 and 24 hours. PBMC-derived monocytes from CL patients (n=05) were infected for 4 and 24 hours with *L.braziliensis* (ratio 5:1) stained with CFSE. (A) TLR2 expression in classical ($CD14^{high}CD16^{-}$), intermediate ($CD14^{high}CD16^{+}$), and non-classical monocytes ($CD14^{low}CD16^{+}$) from CL patients. (B) TLR4 expression in monocytes subsets from CL patients after infection by *L. braziliensis*. Data are represented by the median of the mean intensity of fluorescence (MIF). Mann-Whitney and Kruskal-Wallis with Dunn's post-test was used for statistical test was used for statistical analyses (*P<0.05, **P<0.01, P***<0.001).

Supplementary figure 3. Expression of TNF and IL-10 in TLR 2(+) and TLR4 (+) Monocytes from CL Patients Infected with *L.braziliensis* after 8 and 24 hours. PBMC-derived monocytes from CL patients (n=5) were infected with *L.braziliensis* (ratio 5:1) for 8 and 24 hours. The data represent the frequency of cells expressing TNF **(A)** and IL-10 **(B)** on monocytes expressing $TLR^{(+)}$ and TLR4 $^{(+)}$. The percentage of CD14+ cells expressing cytokines on cells TLR $^{(+)}$ was determined by gating on the corresponding population. The results are expressed as median and Mann-Whitney test was used for statistical analyses (*P<0.05).
